# Comparison of Students’ Physical Activity at Different Times and Establishment of a Regression Model for Smart Fitness Trackers

**DOI:** 10.3390/s25061726

**Published:** 2025-03-11

**Authors:** Xiangrong Cheng, Jingmin Liu, Ye Wang, Yue Wang, Zhengyan Tang, Hao Wang

**Affiliations:** 1 Division of Sports Science and Physical Education, Tsinghua University, Beijing 100084, China; 13969869088@163.com (X.C.); wangye@163.com (Y.W.); wangy@126.com (Y.W.); tzy0565@sina.com (Z.T.); wanghao@163.com (H.W.); 2 Division of Physical Education, Qingdao University of Technology, Qingdao 266520, China

**Keywords:** Chinese students, physical activity, sedentary behavior, smart fitness tracker, step count

## Abstract

Under the strategy of Healthy China, students’ physical health status not only affects their future life and studies but also influences social progress and development. By monitoring and measuring the daily PA levels of Chinese students over a week, this study aimed to fully understand the current PA status of students at different times, providing data support for improving students’ PA levels and physical health. (1) Wearable fitness trackers have advantages such as low cost, portable wearability, and intuitive test data. By exploring the differences between wearable devices and PA testing instruments, this study provides reference data to improve the accuracy of wearable devices and promote the use of fitness trackers instead of triaxial accelerometers, thereby advancing scientific research on PA and the development of mass fitness. A total of 261 students (147 males; 114 females) were randomly selected and wore both the Actigraph GT3X+ triaxial accelerometer and Huawei smart fitness trackers simultaneously to monitor their daily PA levels, energy metabolism, sedentary behavior, and step counts from the trackers over a week. The students’ PA status and living habits were also understood through literature reviews and questionnaire surveys. The validity of the smart fitness trackers was quantitatively analyzed using ActiLife software 6 Data Analysis Software and traditional analysis methods such as MedCal. Paired sample Wilcoxon signed-rank tests and mean absolute error ratio tests were used to assess the validity of the smart fitness trackers relative to the Actigraph GT3X+ triaxial accelerometer. A linear regression model was established to predict the step counts of the Actigraph GT3X+ triaxial accelerometer based on the step counts from the smart fitness trackers, aiming to improve the accuracy of human motion measurement by smart fitness trackers. There were significant differences in moderate-to-high-intensity PA time, energy expenditure, metabolic equivalents, and step counts between males and females (*p <* 0.01), with females having higher values than males in both moderate-to-high-intensity PA time and step counts. Sedentary behavior showed significant differences only on weekdays between males and females (*p <* 0.05), with females engaging in less sedentary behavior than males. (2) There was a significant difference in sedentary time between weekdays and weekends for students (*p <* 0.05), with sedentary time being higher on weekends than on weekdays. (3) Compared with weekends, female students had significantly different moderate-to-high-intensity PA time and sedentary time on weekdays (*p <* 0.01), while no significant differences were observed for male students. (4) Under free-living conditions, the average daily step count monitored by the smart fitness trackers was lower than that measured by the Actigraph GT3X+ triaxial accelerometer, with a significant difference (*p <* 0.01), but both showed a positive correlation (r = 0.727). (5) The linear regression equation established between the step counts monitored by the smart fitness trackers and those by the Actigraph GT3X+ triaxial accelerometer was y = 3677.3157 + 0.6069x. The equation’s R^2^ = 0.625, with an F-test value of *p <* 0.001, indicating a high degree of fit between the step counts recorded by the Huawei fitness tracker and those recorded by the triaxial accelerometer. The *t*-test results for the regression coefficient and constant term were t = 26.4410 and *p <* 0.01, suggesting that both were meaningful. The tested students were able to meet the recommended total amount of moderate-intensity PA for 150 min per week or high-intensity PA for 75 min per week according to the “Chinese Adult PA Guidelines”, as well as the recommended daily step count of more than 6000 steps per day according to the “Chinese Dietary Guidelines”. (2) Female students had significantly more moderate-to-high-intensity PA time than male students, but lower energy expenditure and metabolic equivalents, which may have been related to their lifestyle and types of exercise. On weekends, female students significantly increased their moderate-to-high-intensity PA time compared with males but also showed increased sedentary time exceeding that of males; further investigation is needed to understand the reasons behind these findings. (3) The step counts monitored by the Huawei smart fitness trackers correlated with those measured by the Actigraph GT3X+ triaxial accelerometer, but the step counts from the fitness trackers were lower, indicating that the fitness trackers underestimated PA levels. (4) There was a linear relationship between the Huawei smart fitness trackers and the Actigraph GT3X+ triaxial accelerometer. By using the step counts monitored by the Huawei fitness trackers and the regression equation, it was possible to estimate the activity counts from the Actigraph GT3X+ triaxial accelerometer. Replacing the Actigraph GT3X+ triaxial accelerometer with Huawei smart fitness trackers for step count monitoring significantly reduces testing costs while providing consumers with intuitive data.

## 1. Introduction

In China, diabetes, cardiovascular and cerebrovascular diseases, and obesity are showing a trend towards younger ages. Eighty-five percent of chronic disease patients are between the ages of 14 and 64, belonging to the young and middle-aged working population. Chronic diseases have become the primary health threat to humans in the 21st century [1]. Lack of physical activity is an independent high-risk factor for chronic diseases, increasing the risk of heart disease and diabetes by 20% to 30%. Globally, over 1.4 billion adults face disease risks due to insufficient PA, resulting in an estimated 1.9 million deaths worldwide. Higher physical activity volumes are associated with longer life expectancy, with a higher physical activity intensity profile further adding to a longer life [2]. Currently, according to global data estimates, one-quarter of adults and 81% of adolescents do not engage in sufficient physical exercise [3,4]. Additionally, with the development of national economic zones, changes in transportation methods, and increased work and leisure activities, sedentary behavior accounts for up to 70% of people’s lifestyles. Insufficient PA has negative impacts on society, the economy, the environment, health systems, and people’s quality of life [5]. Researchers, both domestically and internationally, are dedicated to measuring PA levels across various regions and populations to identify and address issues [6,7,8]. Recent monitoring results from China’s Ministry of Education show that the physical fitness of Chinese students is comprehensively declining, with student health issues becoming particularly prominent [9]. As the main force driving societal development, the physical fitness and health status of students are concerning [10]. This study advocates for Chinese students to participate in physical activities based on the World Health Organization’s Global Action Plan for PA (2018–2030), aiming to build a healthy nation and world.

The methods for measuring physical activity include standard methods such as the double-standard water method, indirect calorimetry, and diary recording; subjective methods such as the International Physical Activity Questionnaire, the Global Physical Activity Questionnaire, and the Minnesota Leisure-Time Physical Activity Questionnaire; and objective methods such as the triaxial acceleration sensor method, heart rate belt measurements, and smart fitness tracker measurements [11,12,13]. Although the standard measurement methods have high accuracy, they generally require complex equipment and cumbersome operation. The subjective measurement methods are simple to operate and can measure samples on a large scale, but require professional interviewers to ask questions or manage the process. Objective measurement methods are popular due to their ease of use and high accuracy, but three-axis acceleration sensors have problems such as high cost, unintuitive test data, complex data processing, and insufficient comfort in wearing heart rate band measurement methods [14]. As a wearable device, the motion smart bracelet does not require professional guidance for monitoring and has the advantages of low price, easy to wear, real-time and intuitive data, and suitability for monitoring physical activity in large samples and daily life, and it is of great significance for the public and researchers to monitor physical activity under free-living conditions [15,16]. This study compares a three-axis acceleration sensor and a motion smart bracelet. On the one hand, through the monitoring of a large amount of data, a regression model for the smart motion bracelet was established, forming data conversion between the three-axis acceleration sensor and the motion smart bracelet. This allowed for the collection of physical activity data from subjects in a large area, at low cost, and over a short period of time. On the other hand, for manufacturers developing motion smart bracelets, it can provide effective data to help them improve the accuracy of the monitoring indicators of the motion smart bracelet [17,18,19].

## 2. Research Object and Method

### 2.1. Research Object

This study recruited 261 students as subjects, including 147 males and 114 females. The demographic data are shown in Table 1. All subjects participated voluntarily and were in good health, without any sports injuries or chronic diseases. Before the test, the subjects were informed about the purpose of the test, the testing process, and precautions. Throughout the testing process, the subjects did not change their original lifestyle or habits.

### 2.2. Materials and Method

#### 2.2.1. Materials

The testing instruments used in this study mainly included triaxial accelerometers, fitness trackers, and body composition analyzers. The triaxial accelerometer employed was the Actigraph GT3X+, produced in the United States (hereinafter referred to as the accelerometer). The fitness tracker used was the Huawei 50 m waterproof fitness tracker (hereinafter referred to as the Huawei fitness tracker), and the body composition analyzer used was the Tsinghua Tongfang BCA-2A body composition analyzer.

The core of the accelerometer is a triaxial accelerometer sensor, which converts acceleration or deceleration signals during human activity into electrical signals using electronic principles. These electrical signals are then processed into accelerometer counts, recording the acceleration data in three directions during movement. Using advanced digital filtering algorithms, it measures the PA intensity level, activity count, energy expenditure, and metabolic equivalents of the subject [20].

The Huawei smart fitness tracker (50 m waterproof, launched in 2017, and three-axis acceleration sensor) features intelligent reminders for calls or messages, an intelligent alarm clock, independent GPS (which can provide data such as distance and pace), exercise guidance (using the Firstbeat algorithm to calculate oxygen consumption, provide recovery time suggestions, and generate multiple data analysis reports on training effectiveness), continuous heart rate monitoring, scientific sleep tracking (monitoring different sleep stages including deep sleep, light sleep, rapid eye movement, and night-time wakefulness to assess sleep quality), etc. The “Huawei Fitness Health” app wirelessly transmits data using a smartphone, monitoring real-time data such as steps taken, distance traveled, calories burned, flights climbed, moderate-to-vigorous activity time, heart rate, sleep duration, and sleep quality.

The Tsinghua Tongfang BCA-2A body composition analyzer uses MRI-based Chinese human fat and muscle values, dual-energy water and mass spectrometry to measure body water content, and dual-energy X-ray to measure bone mass. Its weight measurement accuracy is 0.1 kg and body composition measurement accuracy is 0.5% [21]. The primary measurements made by the body composition analyzer are weight and body composition. The subject stands barefoot on the body composition analyzer, with both heels and forefeet placed on the foot electrodes. The hand electrodes are held with both hands, with the thumb and the other four fingers holding onto two electrodes, respectively. The arms are the stretched outwards and apart.

#### 2.2.2. Method

Before wearing the bracelet, the body composition of the subjects was measured. The accelerometer was initialized based on the subject’s height, weight, and age. The accelerometer and the smart fitness bracelet were then issued and worn on the non-dominant wrist simultaneously. The subjects wore them continuously for 24 h under free-living conditions for seven consecutive days. After seven days, the accelerometer and the smart fitness bracelet were collected. As the accelerometer was not waterproof, both bracelets were removed during bathing and swimming, and immediately re-worn after these activities were completed. During the test, the smart fitness bracelet was connected to a mobile app and the daily step count was manually recorded based on the data displayed by the app.

The test indicators included PA indicators (low-, moderate-, and high-intensity PA time; energy expenditure; metabolic equivalents; and steps), sedentary behavior indicators (mainly sedentary time), and body composition indicators (height, weight, and body fat percentage).

### 2.3. Mathematical Statistics

The raw data collected by the accelerometer were downloaded using ActiLife software at a time interval of 10 s. Data were calculated and analyzed for participants who wore the device for at least 4 days per week (including 2 days on the weekend) and wore it for at least 10 h per day. The activity counts derived from acceleration and the corresponding step counts recorded by the smart fitness bracelet were analyzed for the validity of the fitness bracelet using SPSS 26.0 and MedCalc 20.0 analysis methods. Spearman correlation analysis (for non-normally distributed data) and linear regression were used to explore the relationship between the smart fitness bracelet and the triaxial accelerometer. A correlation coefficient (|r|) of ≥0.7 was considered to be a high correlation, 0.4 ≤ |r| < 0.7 was considered to be a moderate correlation, and |r| < 0.4 was considered to be a low correlation. Statistical significance was determined when *p* < 0.05. The paired sample Wilcoxon signed-rank test and mean absolute percentage error (MAPE) were used to test the validity of the step counts recorded by the smart fitness bracelet, analyzing the error between the bracelet and the accelerometer. The MAPE calculation formula is as follows: MAPE = 1/n ∑_(t = 1)^n|(actual value—predicted value)/actual value| × 100%. The smaller the MAPE value, the higher the validity of the step counts recorded by the smart fitness bracelet.

## 3. Results

### 3.1. Comparison Analysis of Measurement Results for PA and Sedentary Behavior

The measurement and statistical data for PA and sedentary behavior are shown in Table 2. The average daily low-intensity PA time for the tested students was 244.66 (54.61) min; the average for boys was 243.94 (59.60) min, and for girls it was 245.62 (47.51) min. There was no significant difference between boys and girls (*p* > 0.05), which was not statistically significant. The average daily moderate-to-high-intensity PA time was 108.33 (33.72) min, with boys averaging 100.32 (28.99) min and girls averaging 118.97 (36.68) min. There was a significant difference between boys and girls (*p <* 0.01), which was statistically significant. The average daily energy expenditure was 361.68 (192.38) kcals, with boys averaging 421.96 (173.11) kcals and girls averaging 281.56 (188.32) kcals. There was a significant difference between boys and girls (*p <* 0.01), which was statistically significant. The average daily metabolic equivalent was 1.25 (0.10) METs, with boys averaging 1.28 (0.09) METs and girls averaging 1.22 (0.11) METs. There was a significant difference between boys and girls (*p <* 0.01), which was statistically significant. The average daily activity count was 7704.11 (2190.12), with boys averaging an activity count of 7350.66 (2040.51) and girls averaging 8173.95 (2303.94). There was a significant difference between boys and girls (*p <* 0.01), which was statistically significant. The average daily sedentary behavior time was 885.50 (134.03) min, with boys averaging 893.40 (150.29) min and girls averaging 875.00 (108.76) min. There was no significant difference between boys and girls (*p* > 0.05), which was not statistically significant.

### 3.2. Comparison Analysis of PA and Sedentary Behavior Measurement Results on School Days and Weekends

Table 3 compares the measurement results of PA, sedentary behavior, and energy expenditure between school days and weekends for students. The results show that the average daily time spent on low-intensity PA during school days was 247.00 (70.42) min, while on weekends it was 240.93 (66.78) min. There was no significant difference between school days and weekends (*p* > 0.05), indicating no statistical significance. The average daily time spent on moderate-to-high-intensity PA during school days was 107.42 (42.52) min, while on weekends it was 110.62 (46.68) min. Again, there was no significant difference between school days and weekends (*p* > 0.05), indicating no statistical significance. The average daily energy expenditure during school days was 359.77 (218.30) min, while on weekends it was 365.32 (218.84) min. There was no significant difference between school days and weekends (*p* > 0.05), indicating no statistical significance. The average daily metabolic equivalent during school days was 1.25 (0.13) METs, while on weekends it was 1.25 (0.12) METs. There was no significant difference between school days and weekends (*p* > 0.05), indicating no statistical significance. The average daily number of counts during school days was 7683.36 (2886.27), while on weekends it was 7767.12 (3183.46). There was no significant difference between school days and weekends (*p* > 0.05), indicating no statistical significance. The average daily sedentary time during school days was 878.63 (215.88) minp, while on weekends it was 907.77 (193.46) minp. There was a significant difference between school days and weekends (*p* < 0.05), indicating a statistical significance.

The PA, sedentary behavior, and energy expenditure measurements for male and female students on weekdays and weekends are shown in Table 4. Male students’ average low-intensity PA time on weekdays was 246.94 (73.60) min per day, and on weekends it was 239.41 (71.47) min per day. There was no significant difference between weekdays and weekends (*p* > 0.05), which was not statistically significant. Female students’ average low-intensity PA time on weekdays was 247.09 (66.25) minutes per day, and on weekends it was 242.93 (60.20) min per day. There was no significant difference between weekdays and weekends (*p* > 0.05), which was not statistically significant. Male students’ average moderate-to-high-intensity PA time on weekdays was 101.33 (38.14) minutes per day, and on weekends it was 99.95 (39.68) min per day. There was no significant difference between weekdays and weekends (*p* > 0.05), which was not statistically significant. Female students’ average moderate-to-high-intensity PA time on weekdays was 115.21 (46.42) min per day, and on weekends it was 124.70 (51.39) min per day. There was a significant difference between weekdays and weekends (*p* < 0.05), which was statistically significant. Male students’ average daily energy expenditure on weekdays was 426.58 (203.14) min, and on weekends it was 423.70 (216.25) minutes. There was no significant difference between weekdays and weekends (*p* > 0.05), which was not statistically significant. Female students’ average daily energy expenditure on weekdays was 274.49 (207.20) min, and on weekends it was 288.31 (197.95) min. There was no significant difference between weekdays and weekends (*p* > 0.05), which was not statistically significant. Male students’ average metabolic equivalent on weekdays was 1.28 (0.12) METs, and on weekends it was 1.28 (0.12) METs. There was no significant difference between weekdays and weekends (*p* > 0.05), which was not statistically significant. Female students’ average metabolic equivalent on weekdays was 1.22 (0.12) METs, and on weekends it was 1.21 (0.11) METs. There was no significant difference between weekdays and weekends (*p* > 0.05), which was not statistically significant. Male students’ average daily counts on weekdays were 7434.24 (2692.15), and on weekends they were 2866.34 (193.69). There was no significant difference between weekdays and weekends (*p* > 0.05), which was not statistically significant. Female students’ average daily counts on weekdays were 8001.40 (3091.13), and on weekends they were 8429.01 (3457.71). There was no significant difference between weekdays and weekends (*p* > 0.05), which was not statistically significant. Male students’ average sedentary time on weekdays was 897.47 (215.78) min, and on weekends it was 896.96 (209.27) min. There was no significant difference between weekdays and weekends (*p* > 0.05), which was not statistically significant. Female students’ average sedentary time on weekdays was 854.58 (213.89) min, and on weekends it was 922.02 (169.95) min. There was a significant difference between weekdays and weekends (*p* < 0.01), which was statistically significant.

Table 5 presents the measured results of PA, sedentary behavior, and energy expenditure among students of different genders on weekdays and weekends. On weekdays, male students spent an average of 246.94 (73.60) min per day on low-intensity PA, while female students spent an average of 247.09 (66.25) min, showing no significant difference between genders (*p* > 0.05), which was not statistically significant. However, male students engaged in an average of 101.33 (38.14) min of moderate-to-high-intensity PA per day compared with 115.21 (46.42) min for female students, indicating a significant difference (*p <* 0.01), which was statistically significant. The average sedentary time for male students was 897.47 (215.78) min per day compared with 854.58 (213.89) min for female students, also showing a significant difference (*p <* 0.01), which was statistically significant. The average daily energy expenditure for male students was 426.58 (203.14) min compared with 274.49 (207.20) min for female students, with a significant difference (*p <* 0.01), which was statistically significant. Male students had an average metabolic equivalent (METs) of 1.28 (0.12) METs per day compared with 1.22 (0.12) METs on weekdays for female students, with a significant difference (*p <* 0.01), which was statistically significant. The average number of counts taken by male students was 7434.24 (2692.15) counts per day compared with 8001.40 (3091.13) counts for female students, also showing a significant difference (*p <* 0.01), which was statistically significant.

On weekends, male students spent an average of 239.41 (71.47) min per day on low-intensity PA compared with 242.93 (60.20) min for female students, showing no significant difference between genders (*p* > 0.05), which was not statistically significant. Male students engaged in an average of 99.95 (39.68) minutes of moderate-to-high-intensity PA per day compared with 124.70 (51.39) min for female students, indicating a significant difference (*p <* 0.01), which was statistically significant. The average sedentary time for male students was 896.96 (209.27) min per day compared with 922.02 (169.95) min for female students, showing no significant difference between genders (*p* > 0.05), which was not statistically significant. The average daily energy expenditure for male students was 423.70 (216.25) min compared with 288.31 (197.95) min for female students, with a significant difference (*p <* 0.01), which was statistically significant. Male students had an average metabolic equivalent (METs) of 1.28 (0.12) METs per day compared with 1.21 (0.11) METs on weekdays for female students, with a significant difference (*p <* 0.01), which was statistically significant. The average number of counts taken by male students was 2866.34 (193.69) counts per day compared with 8429.01 (3457.71) for female students, also showing a significant difference (*p <* 0.01), which was statistically significant.

### 3.3. Validity and Prediction Equation Establishment of Step Count Measurement in Healthy Adults Under Free-Living Conditions Using Smart Fitness Trackers

As the data did not follow a normal distribution, the Wilcoxon signed-rank test for paired samples was used, and the results are shown in Table 6. The minimum value of activity counts monitored by the accelerometer was 1856 steps, the maximum value was 18,287 steps, and the 95% CI of the overall median was (6639, 7101) steps. The average daily step count was 7276 ± 2761 steps. The minimum value of steps monitored by the smart sports bracelet was 377, the maximum value was 24,122, and the overall median 95% CI was (4796, 5531) steps. The average daily step count was 5929 ± 3595 steps. The η^2^ = 0.75, which indicated that the validity reached statistical significance. The Spearman correlation analysis results showed a significant correlation between the activity counts monitored by the accelerometer and the steps monitored by the smart fitness wristband (r = 0.727). The paired Wilcoxon test results showed that *p <* 0.001, indicating statistically significant differences, with significant differences between the results monitored by the accelerometer and the smart fitness wristband; the accelerometer was greater than the fitness wristband. The average mean absolute percentage error (MAPE) of the number of steps monitored by the smart sports bracelet was 55.70%, which may have been due to an insufficient sample size or insufficient exercise volume of the subjects. In future studies, the sample size could be increased and other groups of subjects could be selected for comparison.

This study integrated and analyzed the test data using the steps monitored by the smart fitness wristband as the independent variable and the counts monitored by the accelerometer as the dependent variable to establish a regression equation. The least squares method was used to solve the regression parameters, and the linear regression analysis of the two variables yielded the regression equation of y = 3677.3157 + 0.6069x. The equation’s R^2^ = 0.625, with an F-test value of *p <* 0.001, indicating a linear relationship between the steps recorded by the smart fitness wristband and those recorded by the triaxial accelerometer, with more steps recorded by the wristband corresponding with more counts recorded by the accelerometer. The equation’s fitting degree was high. The *t*-test results for the regression coefficient and constant term were *t* = 26.4410 and *p <* 0.01, indicating that the regression coefficient and constant term were meaningful.

## 4. Discussion

According to the “Chinese Guidelines for PA in Adults”, a total weekly PA of 150 min of moderate intensity or 75 min of high intensity can improve cardiopulmonary function, lower blood pressure and blood sugar levels, increase insulin sensitivity, improve lipid profiles, regulate the endocrine system, enhance bone density, maintain or increase lean body mass, reduce body fat accumulation, and control unhealthy weight gain. The average daily moderate-to-high-intensity PA time among students was 108.33 ± 33.72 min, which met the recommendations in the “Chinese Guidelines for PA in Adults”. The “Chinese Dietary Guidelines for Residents” recommends that adults engage in more than 6000 steps of PA per day. In the monitored data, the overall average number of steps per day was 7704.11 ± 2190.12, with boys averaging 7350.66 ± 2040.51 counts/day and girls averaging 8173.95 ± 2303.94 counts/day, both meeting the recommendations of the “Chinese Dietary Guidelines for Residents”. However, the results of student physical fitness tests have been declining year by year [22,23]. The factors causing this trend are multifaceted; they may be related to students’ lack of exercise focusing on strength and flexibility or their insufficient learning and understanding of test techniques.

There were significant differences in the time of moderate-to-high-intensity activities, step counts, energy consumption, and metabolic equivalents between boys and girls. The time of moderate-to-high-intensity physical activities and step counts of girls were significantly higher than those of boys, but the energy consumption and metabolic equivalents of boys were significantly higher than those of girls. The possible reason is that the girls had a lower muscle content or lower exercise intensity than boys. The number of counts taken by girls was significantly higher than that of boys, which may have been due to the higher activity level or smaller pace of the girls, a result consistent with other examples in the literature [24]. Students’ habits and activities differed between school days and weekends. The latter was more relaxed, allowing for varied activities and rest times for each individual. Previous studies have mostly compared students’ physical activities, lacking research on the physical activities of Chinese students at different times [25,26]. This study divided study days and weekends according to fixed class times and break times, and analyzed whether it was necessary to increase physical activity on weekends. There was no significant difference in low, medium, and high physical activity time between study days and weekends, but sedentary time significantly increased. From the perspective of gender, boys’ physical activity time on weekends was slightly lower than that on school days, but there was no significant difference. Girls’ moderate-to-high-intensity physical activity on weekends significantly increased, and their sedentary time significantly increased. During the survey, we learned that girls would participate in some aerobic dance training on weekends, which may have been one of the reasons for the increase in moderate- and high-intensity activities. The increase in sedentary time and the decrease in low-intensity activities also indicated that the girls were less physically active on weekends than on school days, except for specialized fitness activities. However, for girls, the overall physical activity increased and the energy consumption was also higher than on study days.

The choice of Huawei’s smart sports bracelet was due to the fact that Huawei, as a leading brand in China’s sports bracelet research and development, has strong representativeness. The use of the Huawei 50 m waterproof sports bracelet was because the subjects were likely to engage in activities such as swimming and showering and a waterproof bracelet would not need to be removed, which allowed for a closer approximation of actual PA levels. Additionally, it was affordable. However, this bracelet lacked differentiation for low, medium, and high levels of PA. From the results of the experiment, it could be seen that under free-living conditions, the number of steps measured by the smart sports bracelet moderately correlated with the activity counts from the accelerometer (r = 0.727). However, the number of steps monitored by the smart sports bracelet underestimated the activity count of the accelerometer, with an error rate of 55.70%, which was different from other studies [27]. This result may have been due to the uniqueness of certain samples or possibly because the subjects engaged in activities other than walking and running, such as cycling, which primarily involves lower body movements. As the smart sports bracelet is worn on the wrist, it cannot monitor movements of other parts of the body, whereas the accelerometer can record them. By using both the smart sports bracelet and a triaxial accelerometer to monitor students’ steps and establish a prediction equation, more accurate step counts could be estimated using the smart sports bracelet. This has significance for improving the accuracy of smart sports bracelets and guiding their development direction as well as serving public health and fitness [28,29,30].

## 5. Conclusions

The tested students achieved the recommended total amount of PA per week, which is 150 min of moderate-intensity or 75 min of high-intensity PA according to the “Chinese Adults PA Guidelines”, as well as the recommended daily step count of over 6000 steps for adults according to the “Chinese Dietary Guidelines”.

The time of moderate-to-high-intensity physical activity among female students was significantly higher than that of male students, while the sedentary time was lower. However, the energy expenditure and metabolic equivalent of female students were significantly lower than those of male students. Girls’ moderate-to-high-intensity physical activity significantly increased over the weekend and was higher than that of boys. Their sedentary time significantly increased and exceeded that of boys.

The number of steps monitored by smart fitness trackers correlated with the activity counts from the Actigraph GT3X+ triaxial accelerometer, and the number of steps monitored by the smart fitness trackers was lower.

There was a linear relationship between the smart fitness trackers and the Actigraph GT3X+ triaxial accelerometer. Based on the number of steps monitored by the fitness tracker and the regression equation, the activity counts from the Actigraph GT3X+ triaxial accelerometer could be estimated.

## Figures and Tables

**Table 1:** Demographic statistics of the surveyed population ($\bar{X}$ ± S).

	Number	Age (Years)	Height (cm)	Weight (kg)	Body Fat
Male	147	21.40 ± 3.90	175.89 ± 5.02	70.16 ± 11.04	20.60 ± 5.37
Female	114	21.78 ± 3.29	164.35 ± 5.66	55.54 ± 8.90	27.29 ± 3.73
Total	261	20.99 ± 3.70	171.04 ± 7.78	63.88 ± 12.48	23.45 ± 5.78

**Table 2:** Comparison analysis of measurement results for PA and sedentary behavior ($\bar{X}$ ± S).

	Total	Male	Female	*p*-Value
Low-intensity PA time	244.66 ± 54.61	243.94 ± 59.60	245.62 ± 47.51	0.829
Moderate-to-high-intensity PA time	108.33 ± 33.72	100.32 ± 28.99	118.97 ± 36.68	0.000 **
Energy expenditure	361.68 ± 192.38	421.96 ± 173.11	281.56 ± 188.32	0.000 **
Metabolic equivalent	1.25 ± 0.10	1.28 ± 0.09	1.22 ± 0.11	0.000 **
Activity count	7704.11 ± 2190.1	7350.66 ± 2040.51	8173.95 ± 2303.94	0.011 *
Sedentary behavior	885.50 ± 134.03	893.40 ± 150.29	875.00 ± 108.76	0.328

Note: * indicates *p* < 0.05; ** indicates *p* < 0.01. Units for low-intensity PA time, moderate-to-high-intensity PA time, and sedentary behavior are min/d; energy expenditure is kcals/d; metabolic equivalent is METs/d; activity counts are counts/d, as is the case for the following tables.

**Table 3:** Comparison analysis of PA and sedentary behavior measurement results on school days and weekends ($\bar{X}$ ± S).

	School Days	Weekends	*p*-Value
Low-intensity PA time	247.00 ± 70.42	240.93 ± 66.78	0.153
Moderate-to-high-intensity PA time	107.42 ± 42.52	110.62 ± 46.68	0.252
Energy expenditure	359.77 ± 218.30	365.32 ± 218.84	0.679
Metabolic equivalent	1.25 ± 0.13	1.25 ± 0.12	0.574
Activity count	7683.36 ± 2886.27	7767.12 ± 3183.46	0.659
Sedentary behavior	878.63 ± 215.88	907.77 ± 193.46	0.017 *

Note: * indicates *p* < 0.05.

**Table 4:** Comparison analysis of PA and sedentary behavior measurement results among students of different genders ($\bar{X}$ ± S).

	Male			Female		
	School Days	Weekends	*p*-Value	School Days	Weekends	*p*-Value
Low-intensity PA time	246.94 ± 73.60	239.41 ± 71.47	0.206	247.09 ± 66.25	242.93 ± 60.20	0.489
Moderate-to-high-intensity PA time	101.33 ± 38.14	99.95 ± 39.68	0.662	115.21 ± 46.42	124.70 ± 51.39	0.034 *
Energy expenditure	426.58 ± 203.14	423.70 ± 216.25	0.865	274.49 ± 207.20	288.31 ± 197.95	0.468
Metabolic equivalents	1.28 ± 0.12	1.28 ± 0.12	0.673	1.22 ± 0.12	1.21 ± 0.11	0.604
Activity count	7434.24 ± 2692.15	2866.34 ± 193.69	0.462	8001.40 ± 3091.13	8429.01 ± 3457.71	0.153
Sedentary behavior	897.47 ± 215.78	896.96 ± 209.27	0.993	854.58 ± 213.89	922.02 ± 169.95	0.000 **

Note: * indicates *p* < 0.05; ** indicates *p* < 0.01.

**Table 5:** Comparison and analysis of measurement results of students’ PA, sedentary behavior, and energy expenditure at different times ($\bar{X}$ ± S).

	School Days			Weekends		
	Male	Female	*p*-Value	Male	Female	*p*-Value
Low-intensity PA time	246.94 ± 73.60	247.09 ± 66.25	0.976	239.41 ± 71.47	242.93 ± 60.20	0.601
Moderate-to-high-intensity PA time	101.33 ± 38.14	115.21 ± 46.42	0.000 **	99.95 ± 39.68	124.70 ± 51.39	0.000 **
Energy expenditure	426.58 ± 203.14	274.49 ± 207.20	0.000 **	423.70 ± 216.25	288.31 ± 197.95	0.000 **
Metabolic equivalents	1.28 ± 0.12	1.22 ± 0.12	0.000 **	1.28 ± 0.12	1.21 ± 0.11	0.000 **
Activity count	7434.24 ± 2692.15	8001.40 ± 3091.13	0.005 **	2866.34 ± 193.69	8429.01 ± 3457.71	0.001 **
Sedentary behavior	897.47 ± 215.78	854.58 ± 213.89	0.004 **	896.96 ± 209.27	922.02 ± 169.95	0.196

Note: ** indicates *p* < 0.01.

**Table 6:** Comparison of correlation and differences between accelerometer activity counts and step counts monitored by fitness bands.

Worn Device	Minimum Value	Maximum Value	Mean Value	Standard Deviation	η^2^	95% CI	MAPE (%)	r	*p*-Value
Accelerometer	1856	18,287	7276	2761	0.75	(6639, 7101)		0.727	0.000 **
Fitness Band	377	24,122	5929	3595		(4796, 5531)	55.70		

Note: MAPE is the mean absolute percentage error, ** indicates *p <* 0.01, and r represents the correlation coefficient.

## Data Availability

The datasets generated during and/or analyzed during the current study are available from the corresponding author upon reasonable request.
